# The Global Diet Quality Score is Associated with Higher Nutrient Adequacy, Midupper Arm Circumference, Venous Hemoglobin, and Serum Folate Among Urban and Rural Ethiopian Adults

**DOI:** 10.1093/jn/nxab264

**Published:** 2021-10-23

**Authors:** Sabri Bromage, Christopher T Andersen, Amare W Tadesse, Simone Passarelli, Elena C Hemler, Habtamu Fekadu, Christopher R Sudfeld, Alemayehu Worku, Hanna Berhane, Carolina Batis, Shilpa N Bhupathiraju, Teresa T Fung, Yanping Li, Meir J Stampfer, Megan Deitchler, Walter C Willett, Wafaie W Fawzi

**Affiliations:** Harvard T.H. Chan School of Public Health, Boston, MA, USA; Harvard T.H. Chan School of Public Health, Boston, MA, USA; London School of Hygiene and Tropical Medicine, London, UK; Addis Continental Institute of Public Health, Addis Ababa, Ethiopia; Harvard T.H. Chan School of Public Health, Boston, MA, USA; Harvard T.H. Chan School of Public Health, Boston, MA, USA; Save the Children, Washington, DC, USA; Harvard T.H. Chan School of Public Health, Boston, MA, USA; Addis Continental Institute of Public Health, Addis Ababa, Ethiopia; Addis Continental Institute of Public Health, Addis Ababa, Ethiopia; CONACYT—Health and Nutrition Research Center, National Institute of Public Health, Cuernavaca, Mexico; Harvard T.H. Chan School of Public Health, Boston, MA, USA; Channing Division of Network Medicine, Brigham and Women's Hospital, Boston, MA, USA; Harvard T.H. Chan School of Public Health, Boston, MA, USA; Department of Nutrition, Simmons University, Boston, MA, USA; Harvard T.H. Chan School of Public Health, Boston, MA, USA; Harvard T.H. Chan School of Public Health, Boston, MA, USA; Channing Division of Network Medicine, Brigham and Women's Hospital, Boston, MA, USA; Intake–Center for Dietary Assessment, FHI Solutions, Washington, DC, USA; Harvard T.H. Chan School of Public Health, Boston, MA, USA; Channing Division of Network Medicine, Brigham and Women's Hospital, Boston, MA, USA; Harvard T.H. Chan School of Public Health, Boston, MA, USA

**Keywords:** diet quality metrics, dietary diversity, nutrient adequacy, noncommunicable disease, double burden of malnutrition, nutrition transition, nutritional epidemiology, Ethiopia, sub-Saharan Africa, GDQS

## Abstract

**Background:**

Nutritionally inadequate diets in Ethiopia contribute to a persisting national burden of adult undernutrition, while the prevalence of noncommunicable diseases (NCDs) is rising.

**Objectives:**

To evaluate performance of a novel Global Diet Quality Score (GDQS) in capturing diet quality outcomes among Ethiopian adults.

**Methods:**

We scored the GDQS and a suite of comparison metrics in secondary analyses of FFQ and 24-hour recall (24HR) data from a population-based cross-sectional survey of nonpregnant, nonlactating women of reproductive age and men (15–49 years) in Addis Ababa and 5 predominately rural regions. We evaluated Spearman correlations between metrics and energy-adjusted nutrient adequacy, and associations between metrics and anthropometric/biomarker outcomes in covariate-adjusted regression models.

**Results:**

In the FFQ analysis, correlations between the GDQS and an energy-adjusted aggregate measure of dietary protein, fiber, calcium, iron, zinc, vitamin A, folate, and vitamin B12 adequacy were 0.32 in men and 0.26 in women. GDQS scores were inversely associated with folate deficiency in men and women (GDQS Quintile 5 compared with Quintile 1 OR in women, 0.50; 95% CI: 0.31–0.79); inversely associated with underweight (OR, 0.63; 95% CI: 0.44–0.90), low midupper arm circumference (OR, 0.61; 95% CI: 0.45–0.84), and anemia (OR, 0.59; 95% CI: 0.38–0.91) in women; and positively associated with hypertension in men (OR: 1.77, 95% CI: 1.12–2.80). For comparison, the Minimum Dietary Diversity–Women (MDD-W) was associated more positively (*P* < 0.05) with overall nutrient adequacy in men and women, but also associated with low ferritin in men, overweight/obesity in women, and hypertension in men and women. In the 24HR analysis (restricted to women), the MDD-W was associated more positively (*P* < 0.05) with nutrient adequacy than the GDQS, but also associated with low ferritin, while the GDQS was associated inversely with anemia.

**Conclusions:**

The GDQS performed capably in capturing nutrient adequacy–related outcomes in Ethiopian adults. Prospective studies are warranted to assess the GDQS’ performance in capturing NCD outcomes in sub-Saharan Africa.

## Introduction

Ethiopia has made significant progress towards combating undernutrition in recent decades through extensive economic and agricultural development efforts and nutrition interventions (1). From 2000 to 2016, the national prevalence of anemia among women of reproductive age (WRA) decreased from 33.1% to 23.4% (2), but nonetheless remains highly prevalent, particularly in rural areas and select provinces, and 2025 projections are well above national targets (3, 4). Results of the 2016 National Micronutrient Survey also indicated a moderate to high prevalence of depleted iron stores (10.0%), zinc deficiency (33.8%), vitamin B12 deficiency (15.1%), serum folate deficiency (17.3%), and red blood cell folate deficiency (32%) in WRA (5).

Alongside widespread nutrient inadequacy and undernutrition, Ethiopia faces a steadily rising burden of metabolic disease, especially in urban areas (6). Overweight and obesity (BMI ≥25 kg/m^2^) among urban nonpregnant (NP) WRA nearly doubled from 10.9% in 2000 to 21.4% in 2016 (rural overweight increased from 1.4% to 3.5%) (7, 8); the national prevalence of adult hypertension (≥140 mmHg systolic or ≥90 mmHg diastolic blood pressure) increased from 21.5% in 2000 to 23.6% in 2015; and adult elevated fasting blood glucose (≥7 mmol/l) rose from 2.8% in 2000 to 3.8% in 2014 (9). The 2015 National Noncommmunicable Diseases (NCDs) Stepwise Approach to Surveillance survey also found hypertriglyceridemia and elevated LDL cholesterol in 21.0% and 14.1% of adults, respectively; low HDL cholesterol in 68.7%; and metabolic syndrome [based on the International Diabetes Federation definition (10)] in 8.6% of women and 1.8% of men (11).

Dietary inadequacies are key determinants of malnutrition in Ethiopia. Owing to poverty, recurring drought, and a historical dependence on staple grains, dietary diversity and food security are poor throughout the population (1). Ethiopian adults consume exceedingly low quantities of fruits, vegetables, nuts and seeds, whole grains, PUFAs and omega 3 fatty acids, and calcium for optimal health, and estimated intakes of all of these components are lower than African regional averages for adults; estimated consumption of red meat and milk are less than half the global averages (12). Low consumption of nutrient-rich foods is implicated in the high prevalence of inadequate protein, iron, zinc, and vitamin A intakes (48.5%, 12.9%, 50.4%, and 81.9% of NP WRA nationwide, respectively) (13). National diet quality [as measured by the Alternative Healthy Eating Index–2010 (AHEI-2010), which measures diet-related chronic disease risk] has not changed substantially since 1990 (14), nor has the contribution of diet to age-standardized NCD mortality (24.8% in 1990 to 25.3% in 2013) (15); however, these indicators will likely worsen as Ethiopia's nutrition transition accelerates.

To address persisting undernutrition and monitor emerging dietary contributions to NCDs, countries at an early stage in the nutrition transition will benefit from easily operationalized, food-based metrics for measuring different aspects of diet quality. Given its shifting nutrition and epidemiologic profile, Ethiopia provides a useful context for developing such metrics. In this paper, we conducted a secondary analysis of data from Ethiopian men and women to evaluate the performance of a novel food-based Global Diet Quality Score (GDQS) (16) and compare it with the performance of existing diet metrics.

## Methods

### Study population

We analyzed data from the Anemia Etiology in Ethiopia study (17): a cross-sectional, regionally representative 2-stage stratified sample of 2520 WRA (18) (15–49 years) and 1044 men (15–49 years) living in 6 regions of Ethiopia (Addis Ababa, which is predominantly urban, and 5 predominantly rural regions: Afar, Oromia, Amhara, Tigray, and the Southern Nations, Nationalities, and People's Region). Separate sets of participants were sampled in 2 survey waves [1 conducted in the dry season (February–April 2019) and 1 in the wet season (May–September 2019)]. The survey was approved by the Institutional Review Boards of Harvard T.H. Chan School of Public Health and Addis Continental Institution of Public Health.

### Dietary assessment

Diet was assessed from each participant using 2 tablet-assisted methods [a 24-hour recall (24HR) followed by a quantitative FFQ] programmed in SurveyCTO (Dobility) and administered by trained interviewers (17) (owing to the breadth of the analyses performed in this paper, it was necessary to restrict the analysis of 24HR data to women). Analyses of FFQ and 24HR measurements in women allowed us to examine differences in predictive performance of diet metrics scored using the 2 instruments. Participants were initially asked when they would like to be interviewed (to accommodate their schedules and mitigate the burden of participation) and, in most cases, the FFQ and 24HR were administered on the same day unless participants requested otherwise. Both instruments were piloted in 1 urban and 1 rural *kebele* (ward) prior to the survey. In both seasons, all participants in 1 of 12 randomly selected *kebeles* within each of the 6 regions included in this survey were selected to provide a second 24HR within a median of 3 days (maximum, 6 days) after the first to allow adjustment for within-person variation.

The FFQ was developed based on the list of foods included in the Ethiopian Food Composition Tables (19) following established methods (20), and assessed food consumption primarily at the level of individual ingredients. Average portion sizes were assessed using photographic aids. The FFQ captured a reference period of 7 days and used 7 frequency response categories: never in the past week, 1 time a week, 2–4 times a week, 5–6 times a week, 1 time a day, 2–3 times a day, 4–5 times a day, and ≥6 times a day. The assessment included information on food preparation methods, relative grain content of different consumed flour products, whether different boiled and roasted grains were consumed in whole or split form, and whether animals were consumed in the form of meat and/or various organs. Although 822 unique foods or variants of foods (distinguished by preparation method, grain content, grain processing, and parts of animals) were included in the FFQ, the assessment employed branching logic that only asked about variants of a food if the food was first indicated to be consumed at all (allowing a more efficient assessment without compromising the extensiveness of foods enumerated). Of the 822 foods enumerated, 454 were observed to be consumed at least once by participants in the survey.

The 24HR was tailored to the unique cultural and dietary characteristics of Ethiopia, following guidelines by Gibson and Ferguson (21). The 24HR assessed the consumption of ingredients, as well as many mixed dishes, and was implemented using a multiple-pass method incorporating information on the number of meals at which each food was consumed, the number of servings of each food consumed at each meal, and the average portion size of each consumed food (assessed using photographic aids). A total of 113 distinct foods were reported to be consumed at least once by survey participants. When scoring diet metrics using 24HR data, mixed dishes containing significant proportions of multiple food groups were disaggregated so that fractions of the dish mass counted toward different metric components.

For both instruments, we computed intakes of energy and a set of nutrients considered high priority in low- and middle-income countries (22–25): protein, monounsaturated fat, polyunsaturated fat, saturated fat, fiber, calcium, iron, zinc, vitamin A, folate, and vitamin B12. Nutrient content of foods was determined using the Ethiopian Food Composition Tables (which contain information on both ingredients and mixed dishes) (19), supplemented as needed using data from comparable foods drawn primarily from the Tanzania, Uganda, or USDA tables (26–28). Iron content in teff, the most commonly consumed grain, was adjusted for the influence of soil contamination on iron bioavailability (29).

### Scoring diet metrics

Six metrics were scored in men (using FFQ data) and women [using FFQ and 24HR data; refer to the article by Bromage et al. (16) introducing this Supplemental Issue for information on how these metrics are constructed and scored]:

- *Food-based metrics reflecting overall diet quality*: the Global Diet Quality Score (GDQS) (16) and a Prime Diet Quality Score (PDQS)–like metric (30–32), an adaptation of an earlier metric (the PDQS) from which the GDQS was developed.
- *Food-based metrics reflecting nutrient adequacy*: the GDQS positive submetric (GDQS+) (16), computed using only the healthy GDQS food groups, and the Minimum Dietary Diversity–Women indicator (MDD-W) (33). We acknowledge that the MDD-W was originally intended for use in women only; furthermore, we treated this metric as a continuous variable ranging from 0 to 10, rather than as a binary indicator as it is sometimes used.
- *Metrics reflecting NCD risk*: the GDQS negative submetric (GDQS−) (16), computed using only the unhealthy GDQS food groups, and the AHEI-2010 (34), scored using both food and nutrient components.

Results presented in this paper focus on 3 metrics (the GDQS, MDD-W, and AHEI-2010), while results for the GDQS+, GDQS−, and PDQS-like metric are provided in Supplemental Tables.

### Diet quality outcomes

Nutrient intakes computed using 24HR data were adjusted for within-person variation using variance components estimated from the subset of women for whom 2 recalls were available. This adjustment was performed using the Iowa State University method (35), implemented in the Intake Monitoring Assessment and Planning Program (IMAPP) for all nutrients except vitamin B12 [which was adjusted in SAS version 9.4 using the National Cancer Institute method (36), which can accommodate episodically consumed dietary components].

We estimated energy-adjusted nutrient intakes using the residual method (37). In the FFQ data, we constructed a continuous measure of overall nutrient adequacy based on the number of nutrients (out of 8) meeting age- and sex-specific estimated average requirements (EARs) from the Institute of Medicine (or adequate intake level, in the case of fiber) (38); iron adequacy was defined as ≥50% probability of adequacy based on a lognormal requirement distribution (39). In the 24HR data, overall nutrient adequacy was defined as the mean probability of adequacy of the 8 nutrients based on the full-probability method (39). EARs and requirement distributions for iron and zinc were adjusted to account for absorption characteristics of local diets (39–42). We also created a binary measure of overall nutrient inadequacy using a cutoff of <4 adequate nutrients (out of 8) in the FFQ data or <50% mean probability of adequacy in the 24HR data, as well as energy-adjusted continuous measures of overall nutrient adequacy and binary overall nutrient inadequacy.

In addition to nutrient intake and adequacy outcomes, we assessed anthropometric and biomarker outcomes, including the means of duplicate height, weight, midupper arm circumference (MUAC), and diastolic/systolic blood pressure measurements; venous hemoglobin measured by Hemocue Hb 201+, adjusted for altitude, sex, and smoking status (43); and serum ferritin [adjusted for C-reactive protein using the Biomarkers Reflecting Inflammation and Nutritional Determinants of Anemia method (44)], folate, and vitamin B12, measured at International Clinical Laboratories in Addis Ababa. The following cutoffs were applied to derive binary outcomes:

- Underweight and overweight/obesity: BMI <18.5 kg/m^2^ and ≥25 kg/m^2^, respectively (45).
- Low MUAC: <24.5 cm in women and <25.5 cm in men. These cutoffs resulted in the lowest overall misclassification of underweight BMI in a prior international analysis (46).
- Anemia: <12 g/dl hemoglobin in women and <13 g/dl in men (43).
- Depleted iron stores: ferritin <15 μg/l (47).
- Serum folate deficiency: <3 ng/mL (48).
- Serum vitamin B12 deficiency: <203 pg/mL (49).
- Hypertension: ≥130 mmHg systolic or ≥85 mmHg diastolic blood pressure (50).

### Analysis of metric performance

We evaluated and compared the performance of the different metrics (scored using FFQ data in men and FFQ and 24HR data in women) against diet quality outcomes. Methods involved Spearman correlations between metrics and continuous diet quality outcomes; regression models to determine unadjusted and multivariable (adjusted for potential sociodemographic confounders: age, urban/rural locality, education level, marital status, and occupation) estimated marginal means or ORs for different diet quality outcomes within each metric quintile and in terms of a 1-SD increase in each metric; and statistical comparisons of correlation coefficients, as well as trends in measures of association across quintiles, between pairs of metrics (34, 51). We also examined whether covariate-adjusted associations between metrics and hypertension were robust to further adjustment for BMI. In a regression analysis of 24HR data in women, metrics were analyzed using tertiles due to limited variation in metric scores, except for the GDQS+ and GDQS− submetrics (which were excluded from the regression analysis of 24HR data, given insufficient variation in metric scores).

We excluded women who were currently pregnant or breastfeeding. Within each sex and dietary instrument (FFQ and 24HR), we excluded participants with no reported food intake and with energy intakes <3 or >3 SDs from the mean, to limit the influence of implausible values. Correlation and regression analyses were performed separately in the total population of men and the total population of women. We also performed correlation analyses within subgroups defined by urban compared with rural localities (defined as Addis Ababa compared with all other regions) or dry compared with wet seasons (subgroup analyses were performed separately so that seasons were pooled within urban/rural subgroups, and vice versa), and selected comparisons between groups (52). Given sample size constraints, subgroup analyses in regression models were limited to rural men in an FFQ analysis and (separately) rural women in both FFQ and 24HR analyses.

In interpreting comparative metric performance, we prioritized correlations with energy-adjusted nutrient intakes/adequacy and age-adjusted regression models, and defined a subset of higher-relevance diet quality outcomes in regression models (the continuous measures of energy-adjusted overall nutrient adequacy and anthropometric and biomarker outcomes defined using clinically relevant cutoffs), which were distinguished from lower-relevance outcomes (the binary measure of energy-adjusted overall nutrient inadequacy and continuous anthropometric and biomarker outcomes for which clinically relevant cutoffs exist).

Statistical analyses were performed in R v4.03 (The R Foundation for Statistical Computing, Vienna, Austria) (excluding SAS and IMAPP analyses where noted).

## Results

FFQ data from 976 men and 1604 NP nonlactating (NL) WRA and 24HR data from 1593 NP NL WRA were analyzed in this study. Replicate 24HRs were available from 163 women (73 and 90 in the dry and wet seasons, respectively; 25 and 138 in urban and rural areas, respectively). Ages ranged from 15 to 49 years in both men (median, 34 years) and women (median, 30 years). The numbers of men and women by urban/rural and season subgroups are indicated in **Table 1**. Descriptive statistics on the numbers of FFQs, 24HRs, and replicate 24HRs analyzed by sex, season, and region; prevalences of diet quality outcomes by sex and urban/rural locality; and distributions of food group consumption and metric scores by sex, season, and urban/rural locality for each instrument (FFQ and 24HR) are provided in **Supplemental Tables 1–4**.

**TABLE 1:** Spearman correlations between the GDQS (scored using FFQ data) and energy-adjusted nutrients and clinical measurements among Ethiopian adults

	Total		Dry season		Wet season		Rural		Urban	
Outcome	Men, *n* = 976	Women, *n* = 1604	Men, *n* = 483	Women, *n* = 802	Men, *n* = 493	Women, *n* = 800	Men, *n* = 840	Women, *n* = 1317	Men, *n* = 134	Women, *n* = 287
Calcium	0.17^1^	0.13^1^	0.19^1^	0.19^1^	0.13^2^	0.06	0.16^1^	0.10^1^	0.36^1^	0.30^1^
Fiber	0.20^1^	0.14^1^	0.28^1^	0.16^1^	0.10^3^	0.10^2^	0.16^1^	0.12^1^	0.36^1^	0.19^2^
Folate	0.23^1^	0.21^1^	0.23^1^	0.17^1^	0.23^1^	0.26^1^	0.20^1^	0.22^1^	0.39^1^	0.17^2^
Iron	−0.01	0.01	−0.07	−0.07	0.04	0.10^2^	0.00	0.02	−0.10	0.02
MUFA	0.07^3^	0.15^1^	0.07	0.16^1^	0.06	0.14^1^	0.08^3^	0.12^1^	0.08	0.25^1^
Protein	0.07^3^	0.11^1^	0.02	0.11^2^	0.12^2^	0.11^2^	0.08^3^	0.08^2^	0.11	0.21^1^
PUFA	0.06	0.10^1^	0.00	0.06	0.12^2^	0.14^1^	0.06	0.08^2^	0.10	0.16^2^
SFA	0.01	0.03	0.02	0.06	−0.01	0.01	0.03	0.03	0.04	0.08
Vitamin A	0.14^1^	0.11^1^	0.22^1^	0.12^1^	0.02	0.08^3^	0.13^1^	0.09^1^	0.28^1^	0.17^2^
Vitamin B12	−0.09^2^	0.00	−0.01	0.00	−0.17^1^	−0.08^3^	−0.09^3^	−0.05	0.04	0.15^2^
Zinc	0.04	0.10^1^	0.00	0.07^3^	0.06	0.13^1^	0.04	0.07^3^	0.08	0.24^1^
BMI	−0.01	0.10^1^	−0.05	0.10^2^	0.00	0.09^3^	0.00	0.09^2^	−0.05	0.11
MUAC	0.00	0.10^1^	−0.02	0.09^2^	0.01	0.10^2^	0.01	0.09^1^	−0.04	0.11
Hemoglobin	0.03	0.07^2^	0.02	0.08^3^	0.06	0.08^3^	0.04	0.07^3^	−0.02	0.07
Ferritin	−0.03	0.02	−0.15^2^	−0.05	0.08	0.10	−0.05	0.01	0.11	0.10
Serum folate	0.13^1^	0.20^1^	0.08	0.13^2^	0.18^1^	0.27^1^	0.12^1^	0.19^1^	0.22^3^	0.19^3^
Serum B12	0.07^3^	−0.02	−0.04	−0.12^3^	0.19^1^	0.11^3^	0.06	−0.03	0.22^3^	0.17
Systolic BP	0.11^1^	0.08^2^	0.12^3^	0.07	0.09	0.09^3^	0.13^1^	0.07^2^	−0.03	0.12^3^
Diastolic BP	0.09^2^	0.04	0.09	0.08^3^	0.08	0.01	0.11^2^	0.05	−0.03	0.03

Sample sizes (in parentheses) indicate the number of participants for whom food and nutrient intake data were available (*n* values for other outcomes are provided in subsequent tables). Abbreviations: BP, blood pressure; GDQS, Global Diet Quality Score; MUAC, midupper arm circumference.

1 *P* < 0.001.

2 *P* < 0.01.

3 *P* < 0.05.

### Spearman correlations between the GDQS and energy-adjusted nutrient intakes

In the analysis of FFQ data, the GDQS was significantly (*P* < 0.05) and weakly (0.1 ≥ ρ < 0.3) rank-correlated with energy-adjusted intakes of calcium (men, ρ = 0.17; women, ρ = 0.13), fiber (men, ρ = 0.20; women, ρ = 0.14), folate (men, ρ = 0.23; women, ρ = 0.21), and vitamin A (men, ρ = 0.14; women, ρ = 0.11; Table 1). In women, significant albeit weak correlations were also observed for energy-adjusted monounsaturated fat (ρ = 0.15), polyunsaturated fat (ρ = 0.10), protein (ρ = 0.11), and zinc (ρ = 0.10). The GDQS was nonsignificantly correlated with energy-adjusted iron and saturated fat intakes.

As in the FFQ analysis, the GQDS scored using 24HR data in women was significantly (*P* < 0.05) and weakly (0.1 ≥ ρ < 0.3) correlated with energy-adjusted intakes of calcium (ρ = 0.14), folate (ρ = 0.40), protein (ρ = 0.14), vitamin A (ρ = 0.22), and zinc (ρ = 0.06) and was nonsignificantly associated with energy-adjusted iron intake (*P* ≥ 0.05; **Supplemental Table 5**). Unlike the FFQ analysis, the 24HR analysis indicated no significant correlation between the GDQS and energy-adjusted fiber intake (*P* ≥ 0.05), and indicated negative correlations with energy-adjusted fatty acids (monounsaturated fat: FFQ ρ = 0.15 compared with 24HR ρ = −0.28; polyunsaturated fat: FFQ ρ = 0.10 compared with 24HR ρ = −0.19; saturated fat: FFQ ρ = 0.03 compared with 24HR ρ = −0.11) and vitamin B12 (FFQ ρ = 0.00 compared with 24HR ρ = −0.11). The *P* values were ≥ 0.05 for FFQ saturated fat and vitamin B12 data only.

### Spearman correlations between the GDQS, MDD-W, and AHEI-2010 compared with energy-adjusted overall nutrient adequacy

In the analysis of FFQ data, we observed significant (*P* < 0.05) correlations between the GDQS and energy-adjusted overall nutrient adequacies of ρ = 0.32 in men and ρ = 0.26 in women (**Table 2**). Correlations differed significantly (*P* for difference* <* 0.05) between men and women in rural areas (men ρ = 0.34 compared with women ρ = 0.25), but not in urban areas, the wet season, or dry season (*P* for difference ≥ 0.05). We also observed no differences between urban compared with rural areas or the dry compared with wet seasons in either men or women (*P* for difference* ≥* 0.05). The MDD-W was significantly more strongly correlated than the GDQS (*P* for difference < 0.05) in both women (ρ = 0.46) and men (ρ = 0.48), and the AHEI-2010 was significantly less strongly correlated than the GDQS in both women (ρ = 0.03) and men (ρ = 0.12).

**TABLE 2:** Spearman correlations between the GDQS (scored using FFQ data) and energy-adjusted overall nutrient adequacy among Ethiopian adults

	Total, *n* = 965 men, 1596 women		Dry Season, *n* = 478 men, 795 women		Wet Season, *n* = 487 men, 799 women			Rural, *n* = 832 men, 1311 women		Urban, *n* = 130 men, 285 women		
Sex	ρ	*P*	ρ	*P*	ρ	*P*	*P*-diff (dry vs wet)	ρ	*P*	ρ	*P*	*P*-diff (urban vs rural)
Men	0.32	<0.001	0.31	<0.001	0.32	<0.001	0.432	0.34	<0.001	0.22	0.010	0.086
Women	0.26	<0.001	0.27	<0.001	0.25	<0.001	0.335	0.25	<0.001	0.32	<0.001	0.123
*P*-diff (men vs women)	0.054	—	0.226	—	0.093	—	—	0.013	—	0.156	—	—

Overall nutrient adequacy is defined as the energy-adjusted number of nutrients (out of 8) consumed in adequate amounts. Tests for significance of differences (*P*-diff) between correlations follow the method of Eid, Gollwitzer, and Schmidt for independent samples (52). Abbreviations: GDQS, Global Diet Quality Score.

In the analysis of 24HR data in women, correlations with energy-adjusted overall nutrient adequacy were weak but significant (*P* < 0.05) for the GDQS in the total population (ρ = 0.07), rural areas (*P* = 0.08), dry season (ρ = 0.07), and wet season (ρ = 0.08), but not in urban areas (ρ = 0.02; *P ≥* 0.05; Supplemental Table 5). We observed no differences in correlations between urban compared with rural areas or dry compared with wet seasons (*P* ≥ 0.05). The correlation between the MDD-W and energy-adjusted overall nutrient adequacy in the total population was ρ = 0.23 (*P* < 0.05; *P* for difference with GDQS <0.05), while the AHEI-2010 was not significantly correlated with energy-adjusted overall nutrient adequacy (*P* ≥ 0.05).

Comparisons of correlations between the GDQS and other diet metrics with individual energy-adjusted nutrient intakes and overall nutrient adequacy are presented in **Table 3** and Supplemental Table 5.

**TABLE 3:** Comparison of Spearman correlations between the GDQS, MDD-W, and AHEI-2010 (scored using FFQ data) and energy-adjusted nutrients and clinical measurements among Ethiopian adults

			GDQS	MDD-W		AHEI-2010	
Outcome	Sex	*n*	ρ	ρ	*P*-diff	ρ	*P*-diff
Calcium	Men	976	0.17^1^	0.22^1^	0.057	0.10^2^	0.012^3^
	Women	1604	0.13^1^	0.16^1^	0.229	0.01	<0.001^1^
Fiber intake	Men	976	0.20^1^	0.11^1^	0.001^2^	0.23^1^	0.301
	Women	1604	0.14^1^	0.10^1^	0.069	0.19^1^	0.050
Folate	Men	976	0.23^1^	0.12^1^	<0.001^1^	0.23^1^	0.780
	Women	1604	0.21^1^	0.12^1^	<0.001^1^	0.25^1^	0.173
Iron intake	Men	976	−0.01	0.00	0.864	0.04	0.131
	Women	1604	0.01	−0.02	0.062	0.04	0.135
MUFA	Men	976	0.07^3^	0.30^1^	<0.001^1^	−0.18^1^	<0.001^1^
	Women	1604	0.15^1^	0.36^1^	<0.001^1^	−0.12^1^	<0.001^1^
Protein	Men	976	0.07^3^	0.20^1^	<0.001^1^	−0.21^1^	<0.001^1^
	Women	1604	0.11^1^	0.20^1^	<0.001^1^	−0.11^1^	<0.001^1^
PUFA	Men	976	0.06	0.00	0.008^2^	0.26^1^	<0.001^1^
	Women	1604	0.10^1^	0.03	<0.001^1^	0.26^1^	<0.001^1^
SFA	Men	976	0.01	0.19^1^	<0.001^1^	−0.34^1^	<0.001^1^
	Women	1604	0.03	0.20^1^	<0.001^1^	−0.33^1^	<0.001^1^
Vitamin A	Men	976	0.14^1^	0.09^2^	0.055	−0.01	<0.001^1^
	Women	1604	0.11^1^	0.10^1^	0.622	−0.06^3^	<0.001^1^
Vitamin B12	Men	976	−0.09^2^	0.16^1^	<0.001^1^	−0.42^1^	<0.001^1^
	Women	1604	0.00	0.25^1^	<0.001^1^	−0.39^1^	<0.001^1^
Zinc	Men	976	0.04	0.16^1^	<0.001^1^	−0.15^1^	<0.001^1^
	Women	1604	0.10^1^	0.15^1^	0.002^2^	−0.08^2^	<0.001^1^
ONA	Men	965	0.32^1^	0.48^1^	<0.001^1^	0.12^1^	<0.001^1^
	Women	1596	0.26^1^	0.46^1^	<0.001^1^	0.03	<0.001^1^
BMI	Men	965	−0.01	0.13^1^	<0.001^1^	−0.02	0.801
	Women	1596	0.10^1^	0.16^1^	0.010^3^	0.06^3^	0.230
MUAC	Men	965	0.00	0.14^1^	<0.001^1^	−0.02	0.389
	Women	1596	0.10^1^	0.17^1^	0.001^2^	0.07^2^	0.439
Hemoglobin	Men	883	0.03	0.11^1^	0.010^3^	−0.04	0.018^3^
	Women	1485	0.07^2^	0.10^1^	0.133	0.07^3^	0.655
Ferritin	Men	869	−0.03	0.01	0.089	−0.02	0.906
	Women	782	0.02	−0.04	0.029^3^	0.04	0.413
Serum folate	Men	872	0.13^1^	0.14^1^	0.656	0.12^1^	0.940
	Women	784	0.20^1^	0.21^1^	0.431	0.11^2^	<0.001^1^
Serum B12	Men	872	0.07^3^	0.04	0.447	−0.02	0.014^3^
	Women	785	−0.02	−0.07	0.003^2^	−0.07	0.141
Systolic BP	Men	976	0.11^1^	0.12^1^	0.538	0.04	0.048^3^
	Women	1604	0.08^2^	0.07^2^	0.250	0.06^3^	0.338
Diastolic BP	Men	976	0.09^2^	0.14^1^	0.053	0.05	0.166
	Women	1604	0.04	0.06^3^	0.400	0.02	0.502

ONA measures are energy-adjusted and continuous, showing the number of adequate nutrients out of 8. Abbreviations: AHEI-2010, Alternative Healthy Eating Index–2010; BP, blood pressure; GDQS, Global Diet Quality Score; MDD-W, Minimum Dietary Diversity–Women; MUAC, midupper arm circumference; MUFA, monounsatured fatty acids; ONA, overall nutrient adequacy; PUFA, polyunsatured fatty acids.

1 Statistical significance of correlations, and Wolfe's tests comparing metric-outcome correlations for the GDQS compared with MDD-W and GDQS compared with AHEI-2010 (*P*-diff): *P* < 0.001.

2 Statistical significance of correlations, and Wolfe's tests comparing metric-outcome correlations for the GDQS compared with MDD-W and GDQS compared with AHEI-2010 (*P*-diff): *P* < 0.01.

3 Statistical significance of correlations, and Wolfe's tests comparing metric-outcome correlations for the GDQS compared with MDD-W and GDQS compared with AHEI-2010 (*P*-diff): *P* < 0.05.

### Adjusted regression models of the GDQS compared with diet quality outcomes

In men and women, adjusted for age, urban/rural locality, education level, marital status, and occupation, the GDQS (scored using FFQ data) was significantly (*P* for trend across quintiles < 0.05) associated with higher overall nutrient adequacy [quintile (Q) 1 to Q5 ranges in estimated marginal mean number of nutrients meeting dietary adequacy: men, 3.82 to 5.09; women, 4.33 to 5.22] and lower odds of serum folate deficiency (Q5 compared with Q1 ORs: men, 0.79; women, 0.76); lower odds of underweight and low MUAC in women (Q5 compared with Q1 ORs: men, 0.63; women, 0.84) and anemia in women (Q5 compared with Q1: 0.59); and higher serum vitamin B12 in men (Q5–Q1 range, 302–329 pmol/L; results per 1 SD of GDQS score are presented in **Table 4**; quintile-specific results are presented in **Supplemental Table 6**). Notably, the GDQS was also associated with higher blood pressure in men (Q5–Q1 range in systolic blood pressure: 121.0–125.2 mmHg) and women (Q5–Q1 range: 114.4–118.2 mmHg), and hypertension in men (Q5 compared with Q1 OR: 147). Upon further adjustment for BMI, the GDQS remained significantly associated with hypertension in men (*P*-trend < 0.05) and unassociated in women (*P*-trend ≥ 0.05). Results were similar when data were restricted to rural participants, except that analyses of underweight BMI, as well as hemoglobin and serum B12 levels, did not have significant associations (*P*-trend ≥ 0.05), while the GDQS was associated (*P*-trend < 0.05) with higher odds of overweight in women (Q5 compared with Q1 OR: 1.99; Supplemental Table 6).

**TABLE 4:** Covariate-adjusted associations between the GDQS (scored using FFQ data) and diet quality outcomes among Ethiopian adults

Outcome	Statistic	Sex	*n*	*n*, Cases	Per 1 SD	*P*-trend
ONA, #	EMM (95% CI)	M	976	—	0.41 (0.32–0.50)	<0.001^1^
		F	1604	—	0.32 (0.25–0.39)	<0.001^1^
ONA <4	OR (95% CI)	M	976	309	0.61 (0.52–0.71)	<0.001^1^
		F	1604	402	0.65 (0.57–0.73)	<0.001^1^
BMI, kg/m^2^	EMM (95% CI)	M	965	—	−0.08 (−0.25 to 0.10)	0.592
		F	1596	—	0.35 (0.17–0.53)	0.001^2^
BMI <18.5 kg/m^2^	OR (95% CI)	M	965	354	1.09 (0.95–1.26)	0.284
		F	1596	435	0.88 (0.78–0.99)	0.043^3^
BMI ≥25 kg/m^2^	OR (95% CI)	M	965	67	1.00 (0.76–1.32)	0.651
		F	1596	209	1.19 (1.02–1.40)	0.054
MUAC, cm	EMM (95% CI)	M	965	—	−0.09 (−0.26 to 0.08)	0.532
		F	1596	—	0.31 (0.15–0.48)	0.003^2^
Low MUAC	OR (95% CI)	M	965	566	1.13 (0.98–1.30)	0.120
		F	1596	741	0.84 (0.76–0.93)	0.006^2^
Hemoglobin, g/dL	EMM (95% CI)	M	883	—	0.06 (−0.05 to 0.17)	0.484
		F	1485	—	0.08 (0.01–0.15)	0.034^3^
Anemia	OR (95% CI)	M	883	80	0.81 (0.63–1.04)	0.057
		F	1485	254	0.84 (0.73–0.97)	0.027^3^
Ferritin, ng/mL	EMM (95% CI)	M	869	—	−2.97 (−8.22 to 2.27)	0.290
		F	782	—	0.30 (−3.33 to 3.93)	0.648
Low ferritin	OR (95% CI)	M	869	75	1.34 (1.05–1.73)	0.129
		F	782	173	0.94 (0.79–1.13)	0.829
Serum folate, ng/mL	EMM (95% CI)	M	976	—	0.24 (0.05–0.42)	0.044^3^
		F	1604	—	0.48 (0.21–0.74)	<0.001^1^
Folate deficiency	OR (95% CI)	M	976	569	0.79 (0.68–0.91)	0.010^3^
		F	1604	510	0.76 (0.65–0.89)	<0.001^1^
Serum B12, pmol/L	EMM (95% CI)	M	976	—	15.49 (2.28–28.69)	0.047^3^
		F	1604	—	1.79 (−12.58 to 16.15)	0.478
Serum B12 deficiency	OR (95% CI)	M	976	394	0.91 (0.79–1.04)	0.204
		F	1604	355	1.08 (0.93–1.25)	0.408
Systolic BP, mmHg	EMM (95% CI)	M	976	—	1.45 (0.52–2.37)	0.002^2^
		F	1604	—	1.46 (0.69–2.23)	0.001^2^
Diastolic BP, mmHg	EMM (95% CI)	M	976	—	0.78 (0.16–1.41)	0.019^3^
		F	1604	—	0.53 (0.03–1.03)	0.044^3^
Hypertension	OR (95% CI)	M	976	287	1.27 (1.10–1.46)	0.002^2^
		F	1604	484	1.08 (0.97–1.21)	0.317

Models are adjusted for age, urban/rural locality, education level, marital status, and occupation. ONA measures are energy-adjusted and continuous, showing the number of adequate nutrients out of 8. Abbreviations: BP, blood pressure; EMM, estimated marginal mean; GDQS, Global Diet Quality Score; MUAC, midupper arm circumference; ONA, overall nutrient adequacy.

1 Statistical significance of linear trends across metric quintiles (*P*-trend): *P* < 0.001.

2 Statistical significance of linear trends across metric quintiles (*P*-trend): *P* < 0.01.

3 Statistical significance of linear trends across metric quintiles (*P*-trend): *P* < 0.05.

In the analysis of 24HR data in women, there were fewer significant associations between the GDQS and diet quality outcomes: the GDQS was significantly associated (*P* for trend across tertiles ≥ 0.05) with increased overall nutrient adequacy and decreased odds of anemia across metric tertiles (T) in all women (T3–T1 range in mean probability of overall nutrient adequacy: 57.8%–59.3%; T3 compared with T1 OR for anemia: 0.64) and upon restricting to rural areas (T3–T1 range in overall nutrient adequacy: 57.9%–59.4%; T3 compared with T1 OR for anemia: 0.61); and, unlike in analysis of FFQ data, the GDQS was further associated in rural areas with lower diastolic blood pressure (T3–T1 range: 80.1–78.2 mmHg) and odds of hypertension (T3 compared with T1 OR: 0.70; results per 1 SD of GDQS score are presented in **Table 5**; tertile-specific results are presented in Supplemental Table 6). The significant association with hypertension was robust to further adjustment for BMI.

**TABLE 5:** Covariate-adjusted associations between the GDQS (scored using 24HR data) and diet quality outcomes among Ethiopian women

Outcome	Statistic	Sex	*n*	*n*, Cases	Per 1 SD	*P*-trend
ONA, %	EMM (95% CI)	F	1593	—	0.57 (0.22–0.91)	0.001^1^
ONA <50%	OR (95% CI)	F	1593	211	0.76 (0.65–0.88)	<0.001^2^
BMI, kg/m^2^	EMM (95% CI)	F	1571	—	0.14 (−0.04 to 0.32)	0.279
BMI <18.5 kg/m^2^	OR (95% CI)	F	1571	436	0.94 (0.84–1.06)	0.734
BMI ≥25 kg/m^2^	OR (95% CI)	F	1571	211	1.10 (0.94–1.28)	0.213
MUAC, cm	EMM (95% CI)	F	1571	—	0.11 (−0.06 to 0.27)	0.325
Low MUAC	OR (95% CI)	F	1571	727	0.94 (0.85–1.05)	0.527
Hemoglobin, g/dL	EMM (95% CI)	F	1474	—	0.03 (−0.04 to 0.11)	0.243
Anemia	OR (95% CI)	F	1474	255	0.85 (0.74–0.98)	0.012^2^
Ferritin, ng/mL	EMM (95% CI)	F	762	—	0.40 (−3.19 to 3.99)	0.599
Low ferritin	OR (95% CI)	F	762	167	0.98 (0.82–1.18)	0.818
Serum folate, ng/mL	EMM (95% CI)	F	1593	—	−0.01 (−0.29 to 0.27)	0.489
Folate deficiency	OR (95% CI)	F	1593	494	1.07 (0.92–1.25)	0.237
Serum B12, pmol/L	EMM (95% CI)	F	1593	—	−2.76 (−16.68 to 11.16)	0.467
Serum B12 deficiency	OR (95% CI)	F	1593	345	1.12 (0.97–1.30)	0.105
Systolic BP, mmHg	EMM (95% CI)	F	1593	—	−0.28 (−1.06 to 0.49)	0.134
Diastolic BP, mmHg	EMM (95% CI)	F	1593	—	−0.28 (−0.80 to 0.23)	0.054
Hypertension	OR (95% CI)	F	1593	472	0.99 (0.89–1.11)	0.466

Models are adjusted for age, urban/rural locality, education level, marital status, and occupation. ONA: energy-adjusted continuous measure of overall nutrient adequacy (mean probability of adequacy of 8 nutrients). Abbreviations: BP, blood pressure; EMM, estimated marginal mean; GDQS, Global Diet Quality Score; MUAC, midupper arm circumference; ONA, overall nutrient adequacy; 24HR, 24-hour recall.

1 Statistical significance of linear trends across metric tertiles (*P*-trend): *P* < 0.01.

2 Statistical significance of linear trends across metric tertiles (*P*-trend): *P* < 0.05.

### Comparing adjusted associations between the GDQS, MDD-W, and AHEI-2010 compared with diet quality outcomes

In the FFQ analysis, unlike the GDQS, higher MDD-W scores were significantly associated with lower ferritin levels in men (*P* for trend across quintiles < 0.05); unlike the GDQS, the MDD-W did predict anemia but not serum folate (**Table 6**; **Supplemental Table 7**). Restricting to rural areas, unlike the GDQS, the MDD-W was associated in women with increased blood pressure and odds of hypertension [*P*-trend < 0.05; although upon further adjustment for BMI, the MDD-W was marginally associated (*P*-trend = 0.063)] and was positively associated with low ferritin; however, the MDD-W was also positively associated with lower odds of underweight in women, lower odds of a low MUAC in men, and anemia in men and women (*P*-trend < 0.05; Supplemental Table 7). In both the total population and in rural areas, the MDD-W was more strongly predictive of overall nutrient adequacy than the GDQS (*P* for difference in trends < 0.05; Table 6; Supplemental Table 7). Unlike the GDQS, the AHEI-2010 was not significantly predictive of higher blood pressure or hypertension in the total population or of overweight in rural women (*P*-trend ≥ 0.05), although in rural women the AHEI-2010 was significantly positively associated with increased systolic blood pressure (Supplemental Table 7). Further adjustment for BMI did not produce significant associations between the AHEI-2010 and hypertension in men or women in either the total population or rural subgroup.

**TABLE 6:** Comparison of covariate-adjusted associations between the GDQS, MDD-W, and AHEI-2010 (scored using FFQ data) compared with selected outcomes among Ethiopian adults

Comparison Metric						GDQS		Comparison Metric		
	Outcome	Statistic	Sex	*n*	*n*, Cases	Per 1 SD	*P*-trend	Per 1 SD	*P*-trend	*P*-diff
MDD-W	BMI <18.5 kg/m^2^	OR (95% CI)	M	965	354	1.09 (0.95–1.26)	0.284	0.94 (0.81–1.09)	0.240	0.133
			F	1596	435	0.88 (0.78–0.99)	0.043^1^	0.86 (0.76–0.97)	0.019^1^	0.518
	Low MUAC	OR (95% CI)	M	965	566	1.13 (0.98–1.30)	0.120	0.91 (0.79–1.05)	0.133	0.054
			F	1596	741	0.84 (0.76–0.93)	0.006^2^	0.79 (0.71–0.88)	<0.001^3^	0.281
	Anemia	OR (95% CI)	M	883	80	0.81 (0.63–1.04)	0.057	0.74 (0.57–0.96)	0.012^1^	0.205
			F	1485	254	0.84 (0.73–0.97)	0.027^1^	0.75 (0.64–0.88)	0.002^2^	0.283
	Folate deficiency	OR (95% CI)	M	976	569	0.79 (0.68–0.91)	0.010^1^	0.84 (0.72–0.98)	0.021^1^	0.578
			F	1604	510	0.76 (0.65–0.89)	<0.001^3^	0.77 (0.66–0.90)	0.001^2^	0.748
	Low ferritin	OR (95% CI)	M	869	75	1.34 (1.05–1.73)	0.129	1.35 (1.04–1.76)	0.024^1^	0.628
			F	782	173	0.94 (0.79–1.13)	0.829	1.09 (0.90–1.32)	0.237	0.184
	B12 deficiency	OR (95% CI)	M	976	394	0.91 (0.79–1.04)	0.204	0.96 (0.83–1.11)	0.428	0.402
			F	1604	355	1.08 (0.93–1.25)	0.408	1.12 (0.96–1.31)	0.100	0.334
	ONA, #	EMM (95% CI)	M	976	—	0.41 (0.32–0.50)	<0.001^3^	0.59 (0.51–0.68)	<0.001^3^	0.035^1^
			F	1604	—	0.32 (0.25–0.39)	<0.001^3^	0.57 (0.50–0.64)	<0.001^3^	0.004^2^
	BMI ≥25 kg/m^2^	OR (95% CI)	M	965	67	1.00 (0.76–1.32)	0.651	1.11 (0.84–1.46)	0.469	0.439
			F	1596	209	1.19 (1.02–1.40)	0.054	1.24 (1.06–1.46)	0.033^1^	0.408
	Hypertension	OR (95% CI)	M	976	287	1.27 (1.10–1.46)	0.002^2^	1.19 (1.02–1.38)	0.013^1^	0.403
			F	1604	484	1.08 (0.97–1.21)	0.317	1.12 (0.99–1.25)	0.031^1^	0.292
AHEI-2010	BMI <18.5 kg/m^2^	OR (95% CI)	M	965	354	1.09 (0.95–1.26)	0.284	1.08 (0.94–1.25)	0.198	0.744
			F	1596	435	0.88 (0.78–0.99)	0.043^1^	0.93 (0.83–1.04)	0.100	0.727
	Low MUAC	OR (95% CI)	M	965	566	1.13 (0.98–1.30)	0.120	1.17 (1.02–1.35)	0.009^2^	0.528
			F	1596	741	0.84 (0.76–0.93)	0.006^2^	0.87 (0.78–0.96)	0.029^1^	0.481
	Anemia	OR (95% CI)	M	883	80	0.81 (0.63–1.04)	0.057	0.93 (0.74–1.18)	0.707	0.264
			F	1485	254	0.84 (0.73–0.97)	0.027^1^	0.86 (0.75–0.98)	0.105	0.432
	Folate deficiency	OR (95% CI)	M	976	569	0.79 (0.68–0.91)	0.010^1^	0.74 (0.64–0.86)	<0.001^3^	0.353
			F	1604	510	0.76 (0.65–0.89)	<0.001^3^	0.89 (0.76–1.03)	0.107	0.229
	Low ferritin	OR (95% CI)	M	869	75	1.34 (1.05–1.73)	0.129	1.16 (0.91–1.50)	0.304	0.227
			F	782	173	0.94 (0.79–1.13)	0.829	0.88 (0.74–1.05)	0.357	0.388
	B12 deficiency	OR (95% CI)	M	976	394	0.91 (0.79–1.04)	0.204	1.05 (0.92–1.21)	0.337	0.161
			F	1604	355	1.08 (0.93–1.25)	0.408	1.12 (0.97–1.29)	0.199	0.400
	ONA, #	EMM (95% CI)	M	976	—	0.41 (0.32–0.50)	<0.001^3^	0.15 (0.06–0.24)	0.006^2^	0.016^1^
			F	1604	—	0.32 (0.25–0.39)	<0.001^3^	0.02 (−0.04 to 0.09)	0.618	0.004^2^
	BMI ≥25 kg/m^2^	OR (95% CI)	M	965	67	1.00 (0.76–1.32)	0.651	0.86 (0.65–1.13)	0.176	0.210
			F	1596	209	1.19 (1.02–1.40)	0.054	1.07 (0.91–1.27)	0.427	0.593
	Hypertension	OR (95% CI)	M	976	287	1.27 (1.10–1.46)	0.002^2^	1.16 (1.00–1.34)	0.134	0.353
			F	1604	484	1.08 (0.97–1.21)	0.317	1.07 (0.96–1.20)	0.267	0.651

Excluded from this table: outcomes categorized as lower clinical relevance (the binary measure of energy-adjusted overall nutrient inadequacy and continuous anthropometric and biomarker outcomes); refer to Supplemental Table 7 for expanded results. Models are adjusted for age, urban/rural locality, education level, marital status, and occupation. ONA measures are energy-adjusted and continuous, showing the number of adequate nutrients out of 8. Abbreviations: AHEI-2010, Alternative Healthy Eating Index–2010; EMM, estimated marginal mean; GDQS, Global Diet Quality Score; MDD-W, Minimum Dietary Diversity–Women; MUAC, midupper arm circumference; ONA, overall nutrient adequacy.

1 Statistical significance of linear trends across metric quintiles (*P*-trend), and Wald tests comparing trends between the GDQS and other metrics (*P*-diff): *P* < 0.05.

2 Statistical significance of linear trends across metric quintiles (*P*-trend), and Wald tests comparing trends between the GDQS and other metrics (*P*-diff): *P* < 0.01.

3 Statistical significance of linear trends across metric quintiles (*P*-trend), and Wald tests comparing trends between the GDQS and other metrics (*P*-diff): *P* < 0.001.

In the 24HR analysis in women, unlike the GDQS, the MDD-W was significantly (*P* < 0.05) associated with higher odds of low ferritin and was not associated with lower odds of anemia in either the total or rural population, although the MDD-W was positively associated with higher serum B12 levels in rural areas (**Table 7**; Supplemental Table 7). In the total population, the GDQS was significantly predictive of overall nutrient adequacy, while the MDD-W was not. In rural areas, the GDQS and MDD-W significantly predicted overall nutrient adequacy; unlike the GDQS, the AHEI-2010 did not predict lower odds of hypertension or lower diastolic blood pressure. Further adjustment for BMI did not produce significant associations between metrics and hypertension.

**TABLE 7:** Comparison of statistically significant covariate-adjusted associations between the GDQS, MDD-W, and AHEI-2010 (scored using 24HR data) compared with selected diet quality outcomes among Ethiopian women

Comparison Metric						GDQS		Comparison Metric		
	Outcome	Sex	Statistic	*n*	*n*, Cases	Per 1 SD	*P*-trend	Per 1 SD	*P*-trend	*P*-diff
AHEI-2010	Anemia	F	OR (95% CI)	1474	255	0.85 (0.74–0.98)	0.012^1^	0.86 (0.75–0.99)	0.052	0.342
	Low ferritin	F	OR (95% CI)	762	167	0.98 (0.82–1.18)	0.818	0.82 (0.68–0.99)	0.009^1^	0.167
	B12 deficiency	F	OR (95% CI)	1593	345	1.12 (0.97–1.30)	0.105	1.18 (1.02–1.38)	0.034^1^	0.434
	ONA, %	F	EMM (95% CI)	1593	—	0.57 (0.22–0.91)	0.001^2^	−0.32 (−0.66 to 0.03)	0.361	0.039^1^
MDD-W	Anemia	F	OR (95% CI)	1474	255	0.85 (0.74–0.98)	0.012^1^	0.90 (0.78–1.04)	0.372	0.594
	Low ferritin	F	OR (95% CI)	762	167	0.98 (0.82–1.18)	0.818	1.16 (0.96–1.39)	0.004^1^	0.234
	B12 deficiency	F	OR (95% CI)	1593	345	1.12 (0.97–1.30)	0.105	1.03 (0.89–1.20)	0.85	0.173
	ONA, %	F	EMM (95% CI)	1593	—	0.57 (0.22–0.91)	0.001^2^	1.72 (1.38–2.05)	<0.001^1^	0.020^1^

Excluded from this table are outcomes categorized as lower clinical relevance (the binary measure of energy-adjusted overall nutrient inadequacy and continuous anthropometric and biomarker outcomes), and models in which individual trends for both the GDQS and comparison metric were not significant (*P*-trend ≥ 0.05) for both the AHEI-2010 and MDD-W; refer to Supplemental Table 7 for expanded results. Models are adjusted for age, urban/rural locality, education level, marital status, and occupation. ONA: energy-adjusted continuous measure of overall nutrient adequacy (mean probability of adequacy of 8 nutrients). Abbreviations: 24HR, 24-hour recall; AHEI-2010, Alternative Healthy Eating Index–2010; EMM, estimated marginal mean; GDQS, Global Diet Quality Score; MDD-W, Minimum Dietary Diversity–Women; ONA, overall nutrient adequacy.

1 Statistical significance of linear trends across metric tertiles (*P*-trend), and Wald tests comparing trends between the GDQS and other metrics (*P*-diff): *P* < 0.05.

2 Statistical significance of linear trends across metric tertiles (*P*-trend), and Wald tests comparing trends between the GDQS and other metrics (*P*-diff): *P* < 0.01.

Expanded correlation statistics and comparisons are presented in Supplemental Table 5 and expanded regression statistics and model comparisons are presented in Supplemental Tables 6 and 7, respectively. A summary of results of covariate-adjusted regression analyses is presented in **Supplemental Table 8**.

## Discussion

In an analysis of FFQ data from NP NL WRA and adult men in urban and rural Ethiopia, we found the GDQS significantly and weakly (0.1 ≥ ρ < 0.3) correlated with energy-adjusted intakes of key nutrients and overall nutrient adequacy. In regression models controlling for sociodemographic characteristics, the GDQS was further associated with lower odds of serum folate deficiency in men and women; low MUAC, underweight, and anemia in women; and higher serum vitamin B12 and odds of hypertension in men. An analysis of 24HR data from women produced fewer and more modest associations.

Differences observed in metric-nutrient correlations are attributable to differences in metric construction and scoring. For example, the AHEI-2010’s inclusion of fatty acids and continuous scoring of red and processed meat were reflected in higher correlations than the GDQS with polyunsaturated and saturated fats. While the GDQS’ broad range of fruit and vegetable groups and separate, positively scored liquid oil and fish and shellfish groups were reflected in higher correlations than the MDD-W with fiber, folate, and polyunsaturated and saturated fats, the GDQS’ approach of giving no points to high consumption of high fat dairy and red meat was reflected in lower correlations than the MDD-W with monounsaturated fat, protein, vitamin B12, and zinc.

In a multi-country evaluation of men and rural NP NL WRA in 10 African countries and China and of women in India and Mexico, the GDQS was positively associated with overall nutrient adequacy, with no significant difference with the MDD-W (53–56). One reason for the MDD-W's stronger association with overall nutrient adequacy in the current study is the GDQS’ inclusion of negatively scored food groups, which is intended to capture the diet-related NCD risk but which also contributes some dietary nutrients (the GDQS partly addresses this by giving positive scores to red meat and high fat dairy up to specific consumption thresholds, after which these groups receive 0 points). Secondly, in populations with particularly low dietary diversity (such as in rural Ethiopia), the GDQS’ comparatively nuanced scoring system and food list may be less beneficial for capturing variation in nutrient adequacy and may instead add some noise, while the simpler MDD-W, designed to capture nutrient adequacy in resource-poor settings, may be particularly adept in this regard.

Consistent with the above reasoning, the MDD-W was more frequently associated than the GDQS with anemia and anthropometric indicators of undernutrition in the analysis of FFQ data in this study. However, in analyses of rural men and women in other African countries, we did not find significant differences in associations between either metric compared with low MUAC or anemia, and these metrics also displayed similar associations with low MUAC in an analysis of Indian women (53–55). In 1 secondary analysis of FFQ data from pregnant Tanzanian women (the only prior comparison of the MDD-W and the earlier PDQS metric, from which the GDQS was developed), the PDQS was inversely associated with preterm birth, low birth weight, and fetal loss, while the MDD-W was inversely associated with infants being small for gestational age (32).

Although the MDD-W was not intended to capture the NCD risk, a greater number of positive associations between the MDD-W and NCD outcomes than the GDQS in the current study may highlight an advantage of distinguishing between food scored as healthy and unhealthy in the GDQS. However, although observed associations between metrics and hypertension in the current study were mostly robust to further adjustment for BMI, they were likely subject to confounding by physical activity and residual confounding by socioeconomic status [as was also likely the case in a separate evaluation of the GDQS in predominantly rural women in India (55)]. In a 2007 survey of 1485 rural men and women in Malawi, Rwanda, and Tanzania, fruit and vegetable intake (scored positively in all diet metrics) was inversely associated with systolic and diastolic blood pressure after adjustments for socioeconomic factors, BMI, and physical activity; the mean BMIs were low in both that study (20.9 kg/m^2^ in men and 21.6 kg/m^2^ in women) and the current study (19.9 kg/m^2^ in men and 21.0 kg/m^2^ in women), and BMI was positively associated with hypertension in both studies (57).

Performance of the GDQS and other metrics tabulated using 24HR data was usually poorer than when tabulated using the FFQ, as we have also observed in an analysis of national survey data from Mexico (56). One reason for this was high within-person variation in food consumption (a single recall does not reflect a person's long-term diet, which is more relevant for health). Although we adjusted nutrient intakes for intra-person variation (to better reflect correlations between metrics and estimated usual intakes), we did not attempt to adjust food intake prior to computing metric scores. This approach allowed us to more closely reflect associations that might be observed in 24HR surveys, in which 1 day of recall is often collected from most participants.

Another reason for poorer associations observed in the 24HR analysis was low between-person variation in food consumption. This is illustrated by comparison with an earlier secondary analysis of 24HR data from NP NL women across 6 sub-Saharan African data sets, in which means ± SDs of the FGI-10ER (food group indicator summed from 10 groups, minimum intake 15 g per group, equivalent to the continuous MDD-W used in the current study) ranged from 3.0 ± 0.9 to 4.8 ± 1.2 (range in current study, 2.4 ± 0.8) and correlations between the FGI-10ER and the energy-adjusted mean probability of adequacy ranged from 0.29 to 0.48 (correlation in current study, 0.23) (40). Low between-person variation in the 24HR data is primarily attributable to an extreme lack of dietary diversity in this population (only 113 unique foods were reported to be consumed at least once in the past 24 hours). In addition to attenuating associations, low dietary diversity also limited our ability to obtain robust estimates of within-person variation for certain nutrients (particularly vitamin B12) due to the small number of repeat recalls with non-0 consumption.

Most GDQS-nutrient correlations were weaker in rural than urban participants, and restricting to rural participants produced fewer associations between the GDQS and anthropometric and biochemical outcomes of undernutrition. This is notable because although all undernutrition outcomes were more prevalent in the rural than urban group, the mean GDQS scores were similar, suggesting that urban-rural differences in measures of association were not driven by urban-rural disparities in diet quality but by the fact that poor diet quality is a more predictive determinant of undernutrition in urban areas. Given the context of rapidly urbanizing populations in Ethiopia and sub-Saharan Africa, this reinforces the need for valid diet quality metrics for use in the region.

A strength of this analysis was its scope, comprising men and women in 6 regions and 2 seasons, a comparative analysis of FFQ and 24HR data, and the availability of a range of dietary, anthropometric, and biomarker outcomes against which to evaluate metrics. Limitations included limited data on NCD outcomes (of which only prevalent overweight and hypertension were available) and a low prevalence of overweight (7% in men and 13% in women), which diminished the statistical power to derive associations. The cross-sectional study design also limited our ability to infer causal relations, particularly for NCD outcomes (owing to the latency period between dietary exposures and such outcomes). Furthermore, the number of replicate 24HRs collected from urban women (25) was somewhat small [30 to 40 are recommended per stratum (39)], which may have led to residual within-person variation that weakened associations between metrics and nutrient intakes.

In conclusion, we have found the easy-to-use, food-based GDQS and MDD-W to be good measures of key undernutrition indicators among Ethiopian adults. The MDD-W is simpler to use and was more sensitive to nutrient adequacy than the GDQS, although the latter advantage may only apply to populations consuming extremely monotonous diets (53–56). The GDQS’ differentiation of healthy and unhealthy food groups should render it more suitable than the MDD-W for jointly capturing the NCD risk, as we have found in analyses of national survey data from China and Mexico and in cohort analyses in Mexican and US women (54, 56, 58–60). This should render the GDQS a more useful metric overall as Ethiopia's burden of disease shifts steadily toward NCDs and a more holistic means of measuring diet quality is needed. However, more extensive data, especially from longitudinal studies, are needed to evaluate the GDQS’ sensitivity to NCD outcomes in Ethiopia (and more definitively assess performance of the AHEI-2010, which primarily targets the NCD risk). Given the numerous analyses presented in this paper and the potential for associations to be significant by chance, further research should also endeavor to replicate the findings of the current study.

## Supplementary Material

nxab264_Supplemental_Tables_R2

## Notes

Funding for the research was provided by FHI Solutions, recipient of a Bill & Melinda Gates Foundation grant to support Intake–Center for Dietary Assessment.

Author disclosures: TTF is an Associate Editor of the *Journal of Nutrition* and played no role in the Journal's evaluation of the manuscript. All other authors report no conflicts of interest.

Publication costs for this supplement were funded by the Bill & Melinda Gates Foundation in conjunction with FHI Solutions, recipient of a Bill & Melinda Gates Foundation grant to support Intake–Center for Dietary Assessment. The opinions expressed in this publication are those of the authors and are not attributable to the sponsors or the publisher, Editor, or Editorial Board of the *Journal of Nutrition*. The Supplement Coordinators for the supplement publication were Megan Deitchler, Intake–Center for Dietary Assessment at FHI Solutions, Washington, DC; and Sabri Bromage, Harvard T.H. Chan School of Public Health, Boston, MA.

Supplemental Tables 1–8 are available from the “Supplementary data” link in the online posting of the article and from the same link in the online table of contents at https://academic.oup.com/jn.

The GDQS research initiative was launched by Intake – Center for Dietary Assessment. The research was led by Harvard T.H. Chan School of Public Health, Department of Nutrition and carried out in collaboration with researchers at the National Public Health Institute (INSP), Mexico. Funding for the research was provided by FHI Solutions, recipient of a Bill & Melinda Gates Foundation grant to support Intake – Center for Dietary Assessment.

Abbreviations used: AHEI-2010, Alternative Health Eating Index–2010; EAR, estimated average requirement; GDQS, Global Diet Quality Score; GDQS-−, GDQS negative submetric; GDQS+, GDQS positive submetric; IMAPP, Intake Monitoring Assessment and Planning Program; MDD-W, Minimum Dietary Diversity–Women; MUAC, midupper arm circumference; NCD, noncommunicable disease; NL, nonlactating; NP, nonpregnant; PDQS, Prime Diet Quality Score; Q, quintile; T, tertile; WRA, women of reproductive age; 24HR, 24-hour recall.
